# The ICR96 exon CNV validation series: a resource for orthogonal assessment of exon CNV calling in NGS data

**DOI:** 10.12688/wellcomeopenres.11689.1

**Published:** 2017-05-26

**Authors:** Shazia Mahamdallie, Elise Ruark, Shawn Yost, Emma Ramsay, Imran Uddin, Harriett Wylie, Anna Elliott, Ann Strydom, Anthony Renwick, Sheila Seal, Nazneen Rahman

**Affiliations:** 1 Division of Genetics & Epidemiology, The Institute of Cancer Research, London, SM2 5NG, UK; 2 TGLclinical, The Institute of Cancer Research, London, SM2 5NG, UK; 3 Cancer Genetics Unit, Royal Marsden NHS Foundation Trust, London, SM2 5PT, UK

**Keywords:** CNV calling, next-generation sequencing, NGS, TruSight Cancer, BRCA1, BRCA2, cancer predisposition gene

## Abstract

Detection of deletions and duplications of whole exons (exon CNVs) is a key requirement of genetic testing. Accurate detection of this variant type has proved very challenging in targeted next-generation sequencing (NGS) data, particularly if only a single exon is involved. Many different NGS exon CNV calling methods have been developed over the last five years. Such methods are usually evaluated using simulated and/or in-house data due to a lack of publicly-available datasets with orthogonally generated results. This hinders tool comparisons, transparency and reproducibility. To provide a community resource for assessment of exon CNV calling methods in targeted NGS data, we here present the ICR96 exon CNV validation series. The dataset includes high-quality sequencing data from a targeted NGS assay (the TruSight Cancer Panel) together with Multiplex Ligation-dependent Probe Amplification (MLPA) results for 96 independent samples. 66 samples contain at least one validated exon CNV and 30 samples have validated negative results for exon CNVs in 26 genes. The dataset includes 46 exon CNVs in *BRCA1*, *BRCA2*, *TP53*, *MLH1*, *MSH2*, *MSH6*, *PMS2*, *EPCAM* or *PTEN*, giving excellent representation of the cancer predisposition genes most frequently tested in clinical practice. Moreover, the validated exon CNVs include 25 single exon CNVs, the most difficult type of exon CNV to detect. The FASTQ files for the ICR96 exon CNV validation series can be accessed through the European-Genome phenome Archive (EGA) under the accession number EGAS00001002428.

## Introduction

The use of targeted next-generation sequencing (NGS) in clinical genomics has increased the capacity, throughput and affordability of gene testing ^1– 3^. Use of NGS data in the clinical setting requires comprehensive validation of methods. Ideally, this should include evaluation of the NGS test performance in samples with pre-determined positive and negative results to provide information on sensitivity, specificity and false detection rate.

Deletions and duplications of whole exons, termed ‘exon copy number variants’ or ‘exon CNVs’, are an important class of clinically relevant gene mutations ^4^. Accurate exon CNV detection has proved difficult in targeted NGS data, particularly if only a single exon is affected ^5^. This has led many research and clinical laboratories to either exclude exon CNV detection or to use separate methods for their detection, which can substantially increase the time and cost of tests.

Datasets are available for base substitutions and small insertions and deletions ^6, 7^ and for copy number variants ^6^, but datasets with experimentally validated exon CNV data are not widely available. As a result, methods for detecting exon CNVs in NGS data are usually evaluated using simulated and/or in-house data. This hinders tool comparisons, transparency and reproducibility.

We recently released DECoN ( www.icr.ac.uk/DECoN), a tool optimised to detect exon CNVs in targeted NGS panels in the clinical setting ^8^. During our validation of DECoN performance, we utilised samples with orthogonally generated exon CNV data. This proved extremely valuable in our evaluations and we believe such data will also be highly useful to others. We have therefore put together the ICR96 exon CNV validation series, which we present here. This was undertaken as part of the Transforming Genetic Medicine Initiative (TGMI, www.thetgmi.org), a Wellcome funded initiative which is developing frameworks and resources to facilitate genetic medicine.

The ICR96 exon CNV validation series includes data from 96 samples. Each sample has sequencing data generated using the TruSight Cancer Panel (TSCP) a gene-targeted NGS assay for analysis of cancer predisposition genes ^8– 11^ and Multiplex Ligation-dependent Probe Amplification (MLPA) data ^12^. 66 samples contain at least one validated exon CNV, including 25 single exon CNVs and 30 samples have validated negative results for 26 genes. Of note, the series has excellent representation of the cancer predisposition genes most frequently tested in clinical practice with 46 exon CNVs in one of *BRCA1*, *BRCA2*, *TP53*, *MLH1*, *MSH2*, *MSH6*, *PMS2*, *EPCAM* or *PTEN*.

The ICR96 exon CNV validation series has been extremely helpful in our assessment of exon CNV detection tools, and the comprehensive orthogonal data allows evaluation of sensitivity, specificity and false detection rate. We believe the ICR96 exon CNV validation series could serve as a benchmarking set, particularly for the many clinical and research laboratories now undertaking cancer predisposition gene testing.

## Materials and methods

The data included in this resource were generated from two types of studies. Firstly, through the BOCS, FACT and COG studies, which aimed to discover and characterise disease predisposition genes. All patients gave informed consent for use of their DNA in genetic research. The studies have been approved by the London Multicentre Research Ethics Committee (MREC/01/2/18, MREC/01/2/044, 05/MRE02/17 respectively). Secondly, the data included here was obtained through clinical testing by the TGLclinical laboratory, an ISO 15189 accredited genetic testing laboratory that we run. The consent given from patients tested through TGLclinical includes, as standard, consent for the use of samples for quality-control. It also provides the option of consenting to the use of samples/data in research; all patients whose data was included in the ICR96 series approved this option.

We generated high-quality targeted NGS data for the ICR96 exon CNV validation series using the TruSight Cancer Panel (TSCP) v2 which targets exons from 100 cancer predisposition genes ( Supplementary File 1). We prepared targeted DNA libraries from 50 ng genomic DNA using the TSCP and TruSight Rapid Capture kit (Illumina). We followed the manufacturer’s protocol, with the exception of library enrichment pool complexity, which we performed in 48-plex. We sequenced a final 10 pM pooled library on a HiSeq 2500 platform set in Rapid-run mode following standard protocols: 96-plex pool per flow cell, HiSeq® Rapid SBS Kit v2, 101 bp paired-end dual index run, and onboard clustering using HiSeq® Rapid PE Cluster Kit v2. CASAVA v1.8.1 (Illumina) was used to demultiplex and create FASTQ files per sample from the raw base call files.

All 96 samples also had independently generated exon CNV data available. Standard MLPA and/or MLPA by NGS was performed for one or more of the following 32 genes: *APC, ATM, BAP1, BARD1, BMPR1A, BRCA1, BRCA2, BRIP1, CDH1, CDK4, CDKN2A, CHEK2*, *EPCAM* (exon 9 only) *, FH, MLH1*, *MSH2*, *MSH6*, *MUTYH, NBN, NF1, NSD1, PALB2, PMS2* (excluding exons 12-15), *PTEN, RAD51C, RAD51D, RB1, SDHB, SMAD4, STK11, TP53* and *WT1* ( Supplementary Table 1). The *EZH2* exon 1-20 deletion was identified by comparative genomic hybridisation (CGH) array and was also confirmed by fluorescent *in situ* hybridisation (FISH). For simplicity, we included this one CGH result with the MLPA results.

We provide genomic coordinates in both build 37 and build 38 for all results ( Supplementary Table 1). The genomic coordinates are the most 5’ and most 3’ coordinates of the exons involved in the exon CNV, as determined by MLPA, according to the specified transcript. Of note, these are not the actual breakpoints; neither MLPA nor targeted NGS data can provide breakpoint sequence information for exon CNVs. We provide the MLPA results for all exon CNVs using the following notation “Exon X deletion/duplication” for single exon CNVs and “Exon X-Y deletion/duplication” for exon CNVs involving more than one exon, where X specifies the number of the first exon involved in the exon CNV with respect to the transcript, Y specifies the number of the last exon involved in the exon CNV with respect to the transcript, and deletion or duplication is specified as appropriate. For all genes except *BRCA1* the numbering is consecutive from the first non-coding exon in the transcript. For *BRCA1* we use the conventional clinical numbering system which does not include exon 4.

## Dataset

The ICR96 exon CNV validation series includes samples from 96 individuals. 66 samples contain at least one validated exon CNV and 30 samples have validated negative results for exon CNVs in 26 genes ( Supplementary Table 1). Two of the 66 individuals had an exon CNV in two different genes, such that the dataset includes a total of 68 exon CNVs. This includes 25 single exon CNVs, the most difficult type of exon CNV to detect.

The dataset can be used to evaluate the performance of any tool that aims to detect exon CNVs in NGS data. It has particular utility in validating cancer predisposition gene exon CNV detection. The dataset has excellent representation of the cancer predisposition genes most frequently tested in clinical practice. *BRCA1* and *BRCA2* are particularly well represented, with 15 *BRCA1* exon CNVs and 10 *BRCA2* exon CNVs, of which 11 and four respectively, are single exon CNVs. The 25 *BRCA1* and *BRCA2* exon CNVs include 22 different mutations. We deliberately included, in the same pool, two separate samples with a *BRCA1* exon 13 duplication. This small exon duplication is one of the most common *BRCA1* mutations in the UK ^13^ and hence we wanted to cover the clinical scenario of having two different individuals with this mutation in the same sequencing run. To provide further representation of the cancer predisposition genes most frequently tested in clinical practice, the dataset includes 21 exon CNVs in *MLH1*, *MSH2*, *MSH6*, *PMS2*, *EPCAM*, *PTEN* or *TP53*. Between the two pools, we ensured there was no difference in the representation of exon CNVs in any particular gene or in the proportion of samples without an exon CNV, to minimise potential batch effects ( Table 1).

**Table 1.** MLPA results for each pool in the ICR96 exon CNV validation series.

	Pool 1	Pool 2
Gene	Number of exon CNVs	
*ATM*	1	1
*BRCA1*	7	8
*BRCA2*	5	5
*CHEK2*	2	3
*EPCAM*	0	1
*EZH2*	0	1
*FH*	1	0
*MLH1*	1	0
*MSH2*	4	4
*MSH6*	1	1
*NF1*	1	0
*NSD1*	3	3
*PALB2*	1	0
*PMS2*	3	2
*PTEN*	0	1
*RAD51C*	0	1
*RB1*	1	0
*SDHB*	1	1
*TP53*	1	2
*WT1*	0	1
Total	33	35
	Samples with no exon CNV	
*APC*, *ATM*, *BAP1*, *BARD1*, *BMPR1A*, *BRCA1*, *BRCA2*, *BRIP1*, *CDH1*, *CDK4*, *CDKN2A*, *CHEK2*, *EPCAM*, *MLH1*, *MSH2*, *MSH6*, *MUTYH*, *NBN*, *PALB2*, *PMS2*, *PTEN*, *RAD51C*, *RAD51D*, *SMAD4*, *STK11*, *TP53*	15	15

## Data availability

The data referenced by this article are under copyright with the following copyright statement: Copyright: © 2017 Mahamdallie S et al.

We have deposited the FASTQ files for all 96 samples in the European Genome-phenome archive (EGA). The accession number is EGAS00001002428. Details of how to access the data is available at EGA or from www.icr.ac.uk/icr96.

Researchers and authors that use the ICR96 exon CNV validation series should reference this paper and should include the following acknowledgement: “This study makes use of the ICR96 exon CNV validation series data generated by Professor Nazneen Rahman’s team at The Institute of Cancer Research, London as part of the TGMI”.

## Supplementary Material

**Supplementary File 1. TSCP targeted BED file.**

Targets of the Illumina TruSight Cancer Panel (TSCP) in BED file format.

**Supplementary Table 1. MLPA results for the ICR96 exon CNV validation series.**

Column headings:

**SampleID** – sample ID in the ICR96 exon CNV validation series

**ICR96Pool** – pool in the ICR96 exon CNV validation series

**Gene** – HGNC symbol

**MLPAResult** – the exon CNV result in standard format for clinical reports if an exon CNV was detected, “Normal” = no exon CNV was detected

**ResultType** – “ExonCNV” = an exon CNV was detected, “Normal” = no exon CNV was detected

**ExonCNVType** – “Deletion” = exon CNV was a deletion, “Duplication” = exon CNV was a duplication, blank = no exon CNV was detected

**ExonCNVSize** – “Single” = exon CNV involving only one exon, “Multi” = exon CNV involving more than one exon, blank = no exon CNV was detected

**Chromosome** – chromosome

**5PrimeExon37** – most 5’ genomic coordinate of most 5’ exon in GRCh37

**3PrimeExon37** – most 3’ genomic coordinate of most 3’ exon in GRCh37

**5PrimeExon38** – most 5’ genomic coordinate of most 5’ exon in GRCh38 converted from 5PrimeExon37 using LiftOver ( https://genome.ucsc.edu/cgi-bin/hgLiftOver)

**3PrimeExon38** – most 3’ genomic coordinate of most 3’ exon in GRCh38 converted from 3PrimeExon37 using LiftOver ( https://genome.ucsc.edu/cgi-bin/hgLiftOver)

**ENST65** – the ENST ID from Ensembl v65 used for annotation and genomic coordinates
