# Supplementary material for: The ICR96 exon CNV validation series: a resource for orthogonal assessment of exon CNV calling in NGS data
Source: Wellcome Open Res. 2017 May 26;2:35. [Version 1] doi: 10.12688/wellcomeopenres.11689.1 (PMC5473400; doi:10.12688/wellcomeopenres.11689.1)
Supplement: Peer review history

# Peer review: Referee response for version 1

**DOI:** 10.21956/wellcomeopenres.12629.r23043

**Published:** 2017-06-13

**Authors:** Katharina Wimmer, Gundula Povysil

**Affiliations:** 1 Division of Human Genetics, Innsbruck Medical University, Innsbruck, Austria; 2 Institute of Bioinformatics, Johannes Kepler University Linz, Linz, Austria

This manuscript describes a dataset consisting of massive paralleled sequencing data (FASTQ files) from 96 human DNA samples (ICR96 samples) which are enriched in samples (66/96) with MLPA validated exon CNVs (especially single exon CNVs). The authors used this dataset to evaluate their recently released method DECoN, a CNV detection tool for targeted NGS panel data and provide these data for similar use to other laboratories.

 We evaluated the usability of this dataset with our recently published method panelcn.MOPS. Our data access was processed quickly without problems. Analysis of all 96 samples with panelcn.MOPS revealed that the dataset is less homogenous than the data used in our study comparing panelcn.MOPS to five different CNV detection tools. Compared to the dataset used in our study, more samples and regions of the ICR96 samples were classified as low-quality and low correlation of read counts between test and control samples was observed in many cases. Additional optimization of panelcn.MOPS to the provided dataset was required, showing that any method needs to be adapted to the data to be analyzed.

 Overall, the manuscript clearly describes a very useful dataset for the evaluation of CNV detection in targeted NGS data.

 Minor comments:

 Although it is mentioned briefly in Materials and Methods, the origin of the two different pools should be described again in the Dataset section.

 Since both, GRCh37 and GRCh38, coordinates are provided in Supplementary Table 1, it should be specified which coordinates are given in the BED file that is provided as Supplementary File 1.

 For easier use in method evaluations, it would be helpful to include the exon number used in Supplementary Table 1 also in Supplementary File 1 e.g. writing SDHB.E8.chr1.17345375.17345454 in column 4 instead of SDHB.chr1.17345375.17345454.

We have read this submission. We believe that we have an appropriate level of expertise to confirm that it is of an acceptable scientific standard.

## References

[1] PovysilGTzikaAVogtJHaunschmidVMessiaenLZschockeJKlambauerGHochreiterSWimmerK: panelcn.MOPS: Copy-number detection in targeted NGS panel data for clinical diagnostics. *Hum Mutat*.2017; 10.1002/humu.23237 10.1002/humu.23237 28449315PMC5518446

# Peer review: Referee response for version 1

**DOI:** 10.21956/wellcomeopenres.12629.r23036

**Published:** 2017-06-06

**Authors:** Stewart Payne

**Affiliations:** 1 North West Thames Regional Genetics Service, Northwick Park Hospital, Harrow, UK

This paper describes a resource which is publicly available to laboratories wishing to validate protocols for using NGS data to detect exon CNVs in a clinical setting.  The CNV validation series appears to be soundly designed and internally validated against a dataset generated by MLPA of normal, single exon and multi exon CNVs. The focus of the series is on the genes most commonly implicated in Mendelian cancer predisposition syndromes with data generated using the TruSight Cancer Panel assay.  It is not entirely clear how valid this dataset would be for assessing CNV detection tools for other targeted gene panels or for data generated by other NGS chemistries and/or platforms.  Nevertheless, this paper describes a useful quality and benchmarking resource for clinical laboratories offering testing for cancer predisposition genes, particularly using the TruSight Cancer Panel assay.

I have read this submission. I believe that I have an appropriate level of expertise to confirm that it is of an acceptable scientific standard.
